# Supplementary material for: A Systematic Framework for Collecting Site-Specific Sampling and Survey Data to Support Analyses of Health Impacts from Land-Based Pollution in Low- and Middle-Income Countries
Source: Int J Environ Res Public Health. 2021 Apr 28;18(9):4676. doi: 10.3390/ijerph18094676 (PMC8125743; doi:10.3390/ijerph18094676)
Supplement: Supplementary file 1 [file ijerph-18-04676-s001.zip › ijerph-1177761-supplementary.pdf]

File S1

ASGM Example

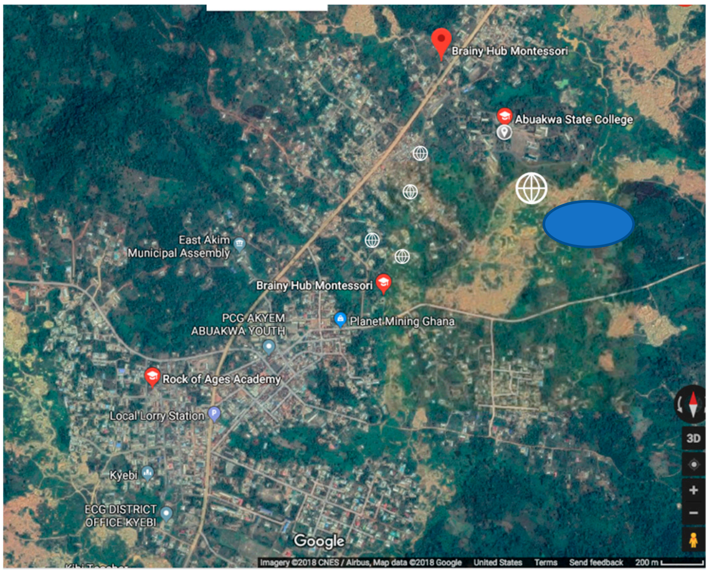

Consider a hypothetical ASGM operation located in a village (population 5800) with the primary ASGM mining area located at the largest white symbol with other known home-based processing areas shown by the smaller white symbols. A small waterbody (blue) is located near the primary processing area, and there is a direct hydrological connection between source area activities and discharges to this water body. The water body may be used as a drinking water source and is a known fishing area for the community. Several schools are downwind of the primary facility, as well as households where auxiliary processing occurs. Children are known to sell lunches at the primary processing area.

ASGM Checklist:

Characterize the general environmental setting on one or more maps:

- Locate ASGM activities in the context of local populations, noting where different aspects of the process may occur. In some areas, grinding and milling occurs in local homes.
- Identify locations of all surface waters, including ditches, creeks, streams, rivers, and lakes.
- Identify what is known about ground water, depth to the water table and aquifers in the study area.
- Identify the prevailing wind direction, particularly relative to residential areas, local waterbodies, and small- or large-scale agricultural activities.
- Identify water bodies within a depositional area of ASGM activities, or impacted by wastewaters or soil runoff. Microbial transformation of mercury to methylmercury and subsequent uptake into aquatic organisms may be an important exposure pathway.
- Identify agricultural areas, community gardens, and the potential for backyard gardening.
- Locate sources of irrigation water that might be impacted by ASGM discharges, including direct or indirect surface water discharges or releases to soils that can runoff or erode. Establish whether ground water is used for irrigation and whether there is a leaching pathway.

Identify locations where animals or animal products (e.g., milk, eggs) are raised for consumption.

Describe the ASGM process:

Identify the source of ores used in the process – lead and arsenic are of concern at ASGM sites.

Calculate the approximate volume (average monthly or annual) of gold production.

Describe the specific ASGM process utilized and identify all inputs and outputs.

Identify the specific amalgamation process (e.g., whole ore or concentrated).

Establish the disposition of mining tailings and note where they are stored/kept. A key concern with ASGM activities is the biotransformation of mercury to methylmercury, particularly in aquatic environments.

Establish where process waters are discharged. A key concern with ASGM activities is the biotransformation of mercury to methylmercury, particularly in aquatic environments.

Waste releases and potential fate and transport:

Develop a qualitative mass balance for ASGM activities by identifying all materials used in the process, where they come from, and what products, including waste, are generated.

Locate wastewater discharges on the site map and identify the specific hydrologic connections between wastewater discharges and surface waters (e.g., ditches, lagoons, receiving waters).

Establish whether typical precipitation events lead to routine ponding and discharges to nearby surface waters with the potential for bioaccumulation into aquatic organisms.

Locate communal surface or ground water sources of drinking water relative to potentially impacted surface waters on the site map to identify potential sampling areas.

Establish the potential for wastewater discharges (directly or indirectly through surface water) to be used as irrigation water for local agricultural products or animals.

In some areas, ASGM processes such as grinding and milling occur in disparate locations, including homes and other public areas removed from areas where amalgamation occurs. These locations need to be identified and waste disposition documented. For example, what happens to the dust generated through these activities?

Population demographics and exposure pathways:

Establish the local population and population size (e.g., village, urban, peri-urban).

Quantify or estimate population size and age/sex distribution.

Identify the fraction of the local population that participates in ASGM activities.

Identify residential areas relative to ASGM activities on the site map.

Identify and map community spaces within the study area, including schools, hospitals and health centers, community centers, places of worship, playgrounds, and places where individuals, particularly children, are likely to spend significant amounts of time.

If processing activities occur in homes (e.g., grinding and milling), these specific locations should be explicitly identified.

Establish site-specific exposure pathways (as shown in Figure 1-2).

Identify whether unique or additional exposure pathways should be considered. Particular emphasis should be given to identifying the sources of drinking water and whether fish consumption occurs, either through recreational angling or commercial operations with fish/seafood going to local markets.

Example ULAB Recycling Facility

Consider a hypothetical ULAB recycling facility (orange triangle) located in a town setting (population 12,000) with some residences located within 100 m of the source. Land use at the site is primarily residential, agricultural, and light industrial. There are three schools (white rectangles) at the site. The source area experiences frequent rain and runoff into the nearby residential area, as well as truck traffic along the road leading into the facility. A number of creeks and ditches are found at the site, and most households obtain drinking water from private or shared wells, while the remaining households obtain their water from a shared surface water source. Nearly half the residents have backyard gardens, and the rest obtain much of their produce and meat from a local market. The green area shows the closest large-scale agricultural field near the ULAB recycling facility. There are also uninhabited areas that have a low potential for exposures to occur.

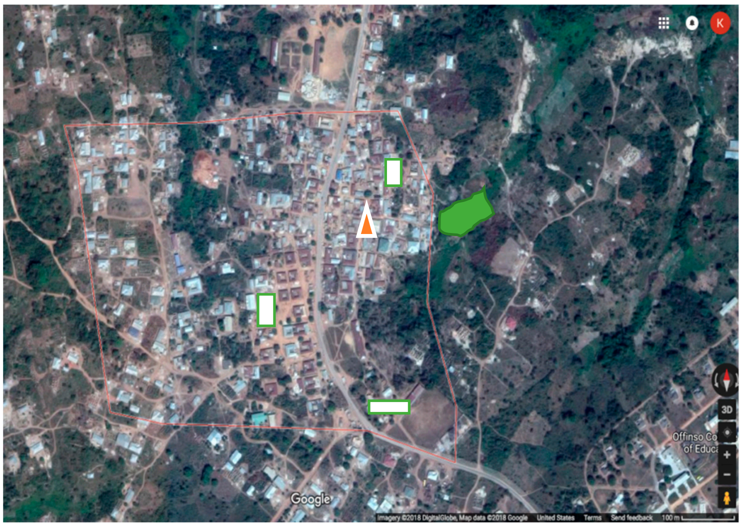

Example: Small-Scale Tannery

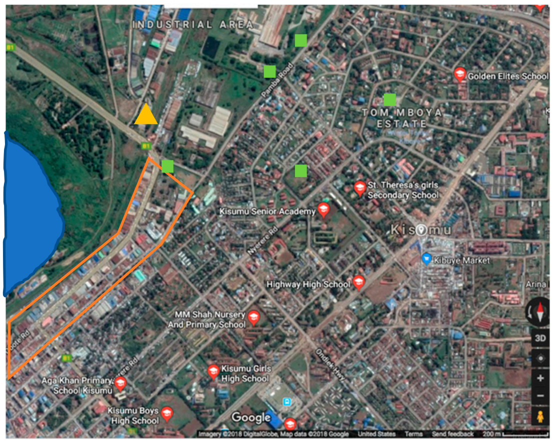

The site is located in a small city (population 25,000) with a lake (outlined in blue) within 1 km of operations. There are 9 schools within several km of the facility. Chickens can be found on the site foraging on onsite solid wastes. In addition, protein and fat materials are separated and used as feed in various locations nearby (depicted by green squares). Various agricultural activities occur within 500 km of the facility, and an agricultural showground is found several km away. Onsite wastewaters discharge into a creek that leads to the lake. Children have been observed playing in the creek and the lake is a popular recreational spot. Municipal water is from ground water, but ground water is only 2-5 m below the surface, thus, contamination from the site is likely. A lower-income area characterized by extremely high population density is found within the orange outline. Municipal water is not available in this area, and residents take water directly from the water body or the various creeks and streams that flow into the lake.
